# One-stage resection of intravascular leiomyomatosis involving the right heart chamber through a single laparotomy

**DOI:** 10.3389/fcvm.2022.976478

**Published:** 2022-10-17

**Authors:** Chaonan Wang, Jiang Shao, Xiao Ma, Yan Zhou, Guotao Ma, Ninghai Cheng, Dongyan Cao, Zhichao Lai, Xitao Song, Kang Li, Bao Liu

**Affiliations:** ^1^Department of Vascular Surgery, Peking Union Medical College Hospital, Peking Union Medical College, Chinese Academy of Medical Sciences, Beijing, China; ^2^National Clinical Research Center for Obstetric & Gynecologic Diseases, Department of Obstetrics and Gynecology, Peking Union Medical College Hospital, Peking Union Medical College, Chinese Academy of Medical Sciences, Beijing, China; ^3^Eight-Year Program of Clinical Medicine, Peking Union Medical College Hospital, Peking Union Medical College, Chinese Academy of Medical Sciences, Beijing, China; ^4^Department of Cardiac Surgery, Peking Union Medical College Hospital, Peking Union Medical College, Chinese Academy of Medical Sciences, Beijing, China

**Keywords:** intracardiac leiomyomatosis, one-stage operation, single laparotomy, technical success, clinical success

## Abstract

**Objectives:**

This retrospective study aimed to summarize the feasibility and experience of utilizing a one-stage operation *via* single laparotomy to treat intracardiac leiomyomatosis (ICL).

**Materials and methods:**

A retrospective study of 13 patients with ICL who underwent one-stage resections was conducted at Peking Union Medical College Hospital from June 2015 to December 2021. All patients had their tumors removed by single laparotomy and were divided into a short venotomy group (6 cases) and an extensive venotomy group (7 cases). We reviewed the patient characteristics, surgical procedures, postoperative pathology, and perioperative and follow-up outcomes of all patients.

**Results:**

All patients underwent surgery for ICL resection using single laparotomy with a 100% success rate. Two patients had tumors distal to the right ventricle (RV), 2 patients had tumors that protruded into the RV in diastole and were confined to the right atrium (RA) in systole, and the other 9 patients had tumors confined to the RA that did not involve the tricuspid valve. The tumor was completely resected in 10 patients, yet 3 patients had a residual tumor. Six patients completed the surgery with short venotomy, 7 completed the surgery with extensive venotomy, and 9 underwent simultaneous total hysterectomy and bilateral adnexal resection. The mean operative time was 370.8 ± 111.0 min, and the mean blood loss was 992.3 ± 994.5 mL. Intraoperative blood loss was lower (483.3 ± 213.7 ml vs. 1429.2 ± 1208.0 ml; P = 0.020) and operative time was shorter (286.5 ± 71.9 min vs. 443.1 ± 84.4 min; P=0.004) in the short venotomy group than in the extensive venotomy group. At a mean follow-up of 26.3 ± 18.8 months, 1 patient had a local recurrence in the pelvis, and 1 patient died of pancreatic cancer, while the remaining patients had no recurrence during follow-up.

**Conclusion:**

One-stage resection of ICL patients by means of a single laparotomy is feasible and effective.

## Introduction

Intravenous leiomyomatosis (IVL) is a rare benign leiomyoma and a specific type of uterine leiomyoma (UM). It usually originates in the uterus and extends to the extrauterine venous system or even the heart and pulmonary arterial system (1, 2). IVL extending into the cardiac cavity is called intracardiac leiomyomatosis (ICL). The current understanding of ICL is mainly based on case reports and case series; its clinical presentation is non-specific and includes right heart obstruction, pulmonary embolism and even sudden death (3). Surgical resection of extrapelvic IVL is the current gold standard treatment, and different surgical centers have reported various surgical strategies (2). At present, consensus on the best surgical strategy for IVL, which simultaneously involves the extrauterine vein, inferior vena cava (IVC) and intracardiac cavity, is lacking. For ICL, the most common surgical procedure is a one-stage sterno-laparotomy under cardiopulmonary bypass (CPB) or even deep hypothermic arrest (DHCA) to remove the intracardiac and intravenous tumor, concurrent hysterectomy and bilateral oophorectomy (4, 5). For certain types of ICLs, a single laparotomy has been used to remove the ICL. Due to the rarity of cases and the heterogeneity of different types of ICLs, consensus on the type of ICL suitable for single laparotomy is lacking, and its clinical effectiveness and prognosis are unclear (6–8).

We describe the resection of ICL using a single laparotomy in 13 patients, either involving the right ventricle (RV) or confined to the right atrium (RA). The clinical features, pathological characteristics, perioperative data, and prognosis of these patients were also retrospectively analyzed.

## Materials and methods

### Patients

Between June 2015 and December 2021, 40 ICL patients underwent surgery at Peking Union Medical College Hospital (PUMCH). Patients were included in the analysis according to the different surgical procedures they underwent, and patients were required to undergo one-stage single laparotomy surgical treatment with the following inclusion criteria: (1) complete perioperative data; (2) confirmed diagnosis on postoperative pathology; (3) complete 12-month follow-up data; (4) no adhesions between the ICL and IVC, endocardium, or heart valves; and (5) the head of the ICL in the heart cavity did not fill the entire heart cavity on preoperative echocardiography. Thus, 27 patients who underwent one-stage sternolaparotomy under CPB or even deep DHCA were excluded, and 13 patients were included in this study. The ethics committee of PUMCH approved the study.

### Demographics

The patients’ clinical features, lesion characteristics, imaging features, and operation procedures were analyzed retrospectively, and postoperative follow-up was carried out (Table 1). The follow-up data were gathered from the outpatient clinic and telephone interviews.

**TABLE 1:** Patient characteristics and preoperative evaluation.

Case (no)	Age (y)	Symptoms	History of hysterectomy	Originated of tumor	Tumor extension	Diameter of the inlet of the RA (mm)	MD of tumor in RA (mm)	MD of IVC infrarenal (mm)	MD of tumor in IVC (mm)
1	50	Mass, dizziness, chest tightness	N	RGV	RA	27	26	21	20
2	36	Abdominal pain	Y	RIIV RCIV	RV	25	7	24	11
3	38	Abdominal pain, chest tightness	N	RGV RIIV RCIV	RV (diastole) RA (systole)	27	12	18	19
4	48	Dizziness, fatigue	Y	RIIV RCIV	RV (diastole) RA (systole)	28	23	25	22
5	46	Abdominal pain	N	RGV RIIV	RV	30	25	31	10
6	54	Abdominal pain, chest tightness, fatigue	N	RIIV RCIV	RA	25	10	22	11
7	48	Mass, palpitation	N	RIIV RCIV	RA	28	13	21	12
8	47	Mass	N	RGV RIIV RCIV	RA	38	22	22	32
9	51	Abdominal pain	Y	RIIV RCIV LGV LIIV	RA	25	17	23	27
10	46	None	N	RIIV RCIV	RA	24	16	23	12
11	48	Limb oedema, chest tightness	Y	LIIV LCIV	RA	46	17	43	45
12	50	Fatigue, limb oedema	N	LGV	RA	28	12	19	25
13	49	Abnormal menstruation	N	RIIV	RA	25	16	14	20

RGV, right gonadal vein; RIIV, right internal iliac vein; RCIV, right common iliac vein; LGV, left gonadal vein; LIIV, left internal iliac vein; LCIV, left common iliac vein; RA, right atrium; RV; right ventricle; IVC, inferior vena cava; MD, Maximum diameter.

According to preoperative examination, all patients in this study were diagnosed with UM. The patients were diagnosed with ICL if postoperative pathology showed that the tumor originated in the uterus and grew into a vein with involvement of the intracardiac cavity.

### Preoperative imaging assessment

**FIGURE 1:**
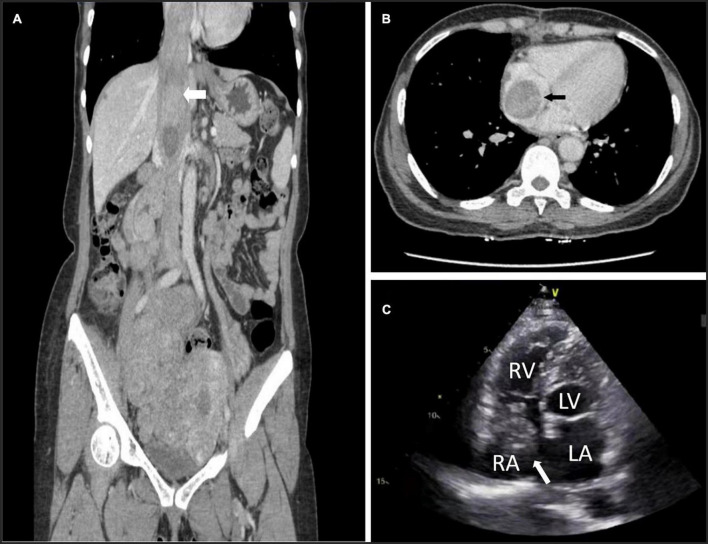
Preoperative computed tomography venography (CTV) and echocardiography can detect tumors. **(A)** Image of patient 5 showing intracardiac leiomyomatosis (ICL) extending through the inferior vena cava (IVC) to the right atrium (RA) (white arrow). **(B)** Image of patient 3 showing a tumor within the RA (black arrow). **(C)** Echocardiogram of patient 10 shows a solid intracardiac head. LA, left atrium (white arrow); LV, left ventricle; RV, right ventricle.

All patients underwent computed tomography venography (CTV), echocardiography, and vascular ultrasound preoperatively to evaluate the tumor’s location, extent, diameter, mobility and extrapelvic metastases (Figure 1). The imaging data measured before the operation included right atrial (RA) inlet, infrarenal IVC maximum diameter, and the maximum diameter of tumors in the RA and IVC.

### Surgical planning

According to the degree of tumor progression, this group of patients was categorized as stage III (9); the tumor reached the level of the renal vein and IVC and further extended to the RA but did not reach the pulmonary artery. Through a multidisciplinary team (MDT) discussion, combined with the imaging findings of the patients, all patients were scheduled for one-stage resection of tumors in the vena cava system, total hysterectomy, and bilateral adnexal resection.

### Surgical approach

**FIGURE 2:**
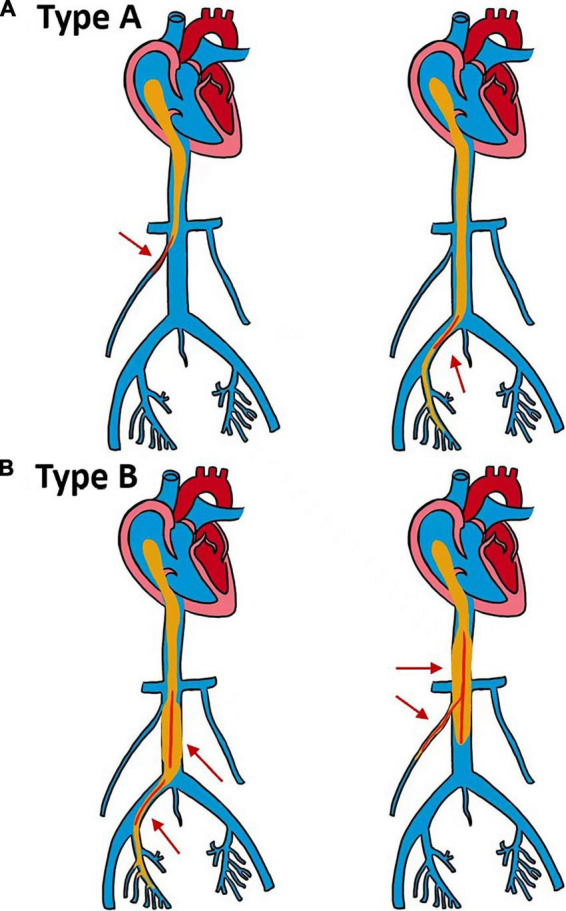
Schematic diagram of two different types of surgery. **(A,B)** The maximum diameter of the intracardiac tumor is smaller than the diameter of the inlet of the right atrium (RA). **(A)** The maximum diameter of the tumor in the inferior vena cava (IVC) is smaller than the maximum diameter of the infrarenal IVC, and the tumor can be removed by a short venotomy (red arrow). **(B)** The maximum diameter of the tumor in the IVC is larger than the maximum diameter of the infrarenal IVC, and extensive venotomy is required to remove the tumor (red arrow).

As shown in Figure 2, we divided 13 patients into types A and B according to the size of the tumor and the degree of involvement. Type A patients underwent short venotomy. Type B patients underwent extensive venotomy. The midline incision began at the pubic symphysis and extended to the umbilicus or the xiphoid, depending on the site of the venous incision.

### Surgical procedure

Bilateral D-J catheters were implanted before the operation, surgery was performed under general anesthesia in the supine position, and transesophageal echocardiography (TEE) was performed to determine the condition of the tumor in the IVC and RA. A longitudinal midline incision was made to expose the tumor, uterus, bilateral ovaries, and fallopian tubes. First, hysterectomy and bilateral salpingo-oophorectomy were performed by gynecologists. According to the source of the tumor and the range of the vena cava, the infrarenal IVC, iliac vein and ovarian vein should be exposed and controlled. If IVL does not invade the gonadal vein, distal transection, and ligation can be performed. If the IVL simultaneously entered into the IVC through the internal iliac vein (IIV), the segment of the common iliac vein (CIV) needed to be incised at the same time. If the maximum diameter of the IVL was more extensive than that of the infrarenal IVC and it was not easy to pull out through the infrarenal incision, an extensive incision of the IVC was needed. Most tumors do not densely adhere to the lumen wall, and once the tumor tissue can be moved in the lumen, the tumor can be removed directly from the RA and gently retrograde pulled from the incision. TEE was used to observe the proximal end of the tumor and to ensure that the tumor was completely removed.

**FIGURE 3:**
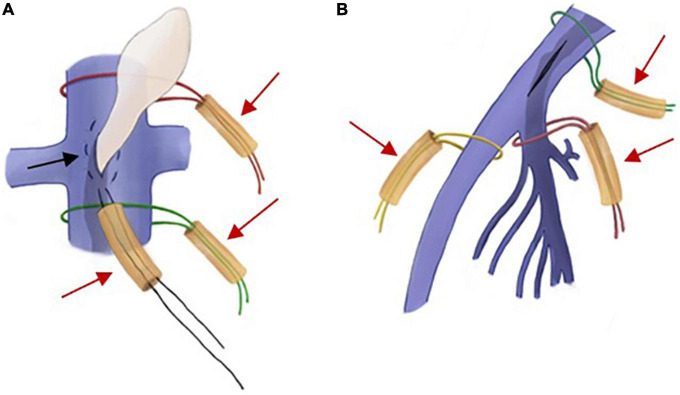
Intraoperative schematic diagram of two different ways of blocking blood flow. **(A)** If the incision is in the inferior vena cava (IVC), a purse-string suture (black arrow) is placed between the proximal and distal blocking bands (red arrow), and the tumor can be dragged out through the incision within the suture. **(B)** If the incision is in the iliac vein, separate blocks are made in the common iliac vein (CIV), the internal iliac vein (IIV), and the external iliac vein (EIV) (red arrows).

During the operation, two different methods were used to block blood flow according to the IVC incision location (Figure 3). For the incision of IVC at the bilateral renal vein level, the IVC, renal vein and iliac vein should be fully exposed at first. Blocking bands covered with a silicone tube were used to block the proximal and distal ends of the IVC. Then, a purse-string 5-0 Prolene suture was placed in the incision, and the suture was tightened with a silicone tube. When the tumor passed through the incision, the 5-0 Prolene suture was further tightened and sutured after the tumor was completely pulled out to suppress bleeding. For the iliac vein incision, the internal and external iliac veins should be fully dissected, and a blocking band covered by a silicone tube should be added to the distal end of the CIV and the proximal end of the internal and external iliac veins.

### Postoperative pathology

**FIGURE 4:**
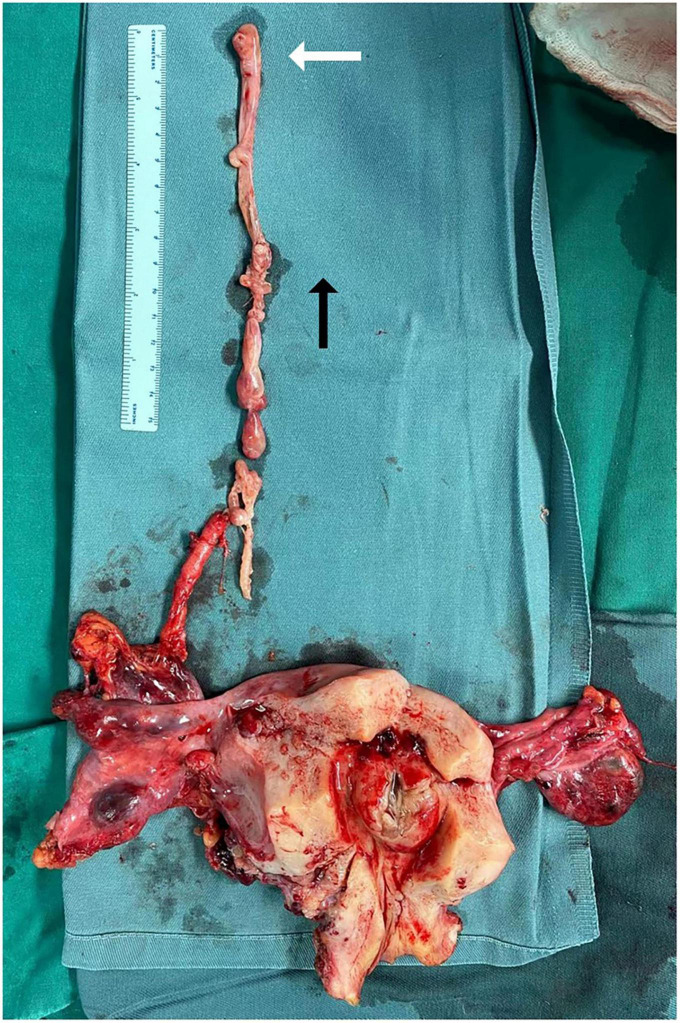
Pathological specimens of intracardiac leiomyomatosis (ICL). The white arrow points to a tumor in the right atrium. Black arrow points to the side of the cranial.

All resected tumor specimens were examined by postoperative pathological examination according to the two appendages of the uterus, the mass of the IVC and the mass of the ovarian veins. The surgical specimens of the patients were fixed with 4% formaldehyde. Paraffin-embedded sections were cut into 3∼4 μm sections and stained with HE (hematoxylin-eosin) and immunohistochemical techniques using the EnVision method. Antibodies against the following were used: SMA, ER, PR, Desmin, CyclinD1, Bcl-2, CD10, and Ki-67. Smooth muscle markers and estrogen and progesterone receptor markers were selected according to previous reports (9). A postoperative pathologic specimen is shown in Figure 4.

### Follow-up

All patients were followed up for 3, 6, and 12 months after the operation and yearly thereafter. After the operations, physical examination, echocardiography, pelvic ultrasound, and CTV examination of the IVC were performed. The primary endpoint of follow-up was complications and death, and the secondary endpoint was local recurrence.

### Statistical analysis

Retrospective data analyses were performed. For all continuous variables, normally distributed variables are represented by the mean ± standard deviation (SD), and the median represents non-normally distributed variables. Categorical data are presented as percentages. Follow-up results were analyzed regarding related recurrence and complications. A *P* < 0.05 was deemed to be statistically significant.

## Results

### Patients

This study included 13 (100%) patients (13 female; mean 43 ± 7.1 years) who underwent a single laparotomy for ICL at PUMCH. All patients had a previous history of reproductive and uterine fibroids (100%), of which 4 patients (30.7%) had undergone hysterectomy and bilateral adnexectomy before hospitalization, and the remaining patients (69.2%) had uterine fibroids on admission.

The patients’ symptoms were different: 7 patients (53.8%) had abdominal pain, and most of the patients presented with multiple symptoms, which primarily included dizziness, palpitation, chest tightness, fatigue, bilateral lower limb edema, pelvic mass, and abnormal menstruation. No patients had severe symptoms, such as venous thromboembolism, heart failure, kidney failure, or respiratory insufficiency.

### Diagnostic results

Preoperative imaging examination can help evaluate the tumor’s location, extent, diameter, mobility, and extrapelvic metastases. According to the results of preoperative imaging examinations, the tumors originated from the right gonadal vein in 5 patients (1, 3, 5, 8, and 9), the right IIV in 10 patients (2, 3, 4, 5, 6, 7, 8, 9, 10, and 13), the right CIV in 8 patients (2, 3, 4, 6, 7, 8, 9, and 10), left gonadal vein in 2 patients (9 and 12), left IIV and CIV in 2 patients (9 and 11); 5 patients’ tumors (3, 5, 8, 9, and 10) originated from the gonadal vein, IIV and CIV at the same time. Combining the results of echocardiography, vascular ultrasound and CTV, we observed a low-medium echogenicity cord-like structural mass in the IVC. The distal end of the tumor was located in the RV in 2 patients (2 and 5); the tumor in the RA could protrude into the RV in diastole and was confined to the RA in systole in 2 patients (3 and 4); the remaining 9 patients (1, 6, 7, 8, 9, 10, 11, 12, and 13) had a mobile solid tumor in the RA that did not involve the tricuspid valve.

The diameter of the RA inlet was 28.9 ± 6.2 mm; the maximum diameter of the tumor within the RA was 16.6 ± 5.9 mm; and the maximum width of the infrarenal IVC was 23.5 ± 7.1 mm. The maximum width of the tumor within the IVC was 21.0 ± 10.3 mm. In six patients (3, 8, 9, 11, 12, and 13), the maximum diameter of the tumor exceeded the infrarenal diameter of the IVC.

### Surgical results

All patients (100%) underwent a successful operation, 9 patients (Nos. 1, 3, 5, 6, 7, 8, 10, and 12) underwent bilateral ligation of the IIV and ovarian vein, hysterectomy and bilateral adnexectomy, and the remaining 4 patients (Nos. 2, 4, 9, and 11) had previously undergone this procedure. Retrograde ICL extraction was performed from the IVC and iliac veins through IVC or iliac venotomy in 13 patients (100%). The preoperative examination results showed that 7 patients (Nos. 1, 2, 4, 5, 6, 7, and 10) were Type A, and 6 patients (Nos. 3, 8, 9, 11, 12, and 13) were Type B. Among them, Type B patients were treated with extensive venotomy, and Type A patients were treated with a short venotomy of the infrarenal IVC or CIV (Table 2).

**TABLE 2:** Surgical procedures and outcomes.

Case	Surgical procedure	Uterus and adnexa	Residual tumor	Incision	Follow-up (months)
1	Type A	Resection	None	IVC	66
2	Type A	/	Tricuspid valve	IVC, RCIV	50
3	Type B	Resection	None	IVC, LCIV	12
4	Type A	/	None	IVC	16
5	Type A	Resection	None	IVC	42
6	Type A	Resection	None	IVC	43
7	Type A	Resection	None	RCIV	36
8	Type B	Resection	Pelvic	IVC, RCIV	5
9	Type B	/	Pelvic	IVC, RCIV	18
10	Type A	Resection	None	RCIV	16
11	Type B	/	None	IVC, LCIV	14
12	Type B	Resection	None	Supra and infra renal IVC	12
13	Type B	Resection	None	IVC, RCIV	12

RCIV, right common iliac vein; LCIV, left common iliac vein; IVC, inferior vena cava.

Since the tumor of 1 patient (No. 2) involved the RV, a residual tumor embolism of approximately 3 mm was observed in the chordae tendineae of the tricuspid valve, and TEE showed that the opening and closing of the tricuspid valve were not disturbed and there was no regurgitation. Thus, this residual tumor was not treated. The postoperative echocardiogram showed a mass of echogenicity at the tricuspid valve chordae tendineae with a size of approximately 5 mm*10 mm, no abnormalities in the morphology and opening and closing of the valve, and a tricuspid regurgitation velocity of 2.4 m/s.

Due to the proximity to the iliac vessels, the complete removal of the tumor was difficult, and 2 patients (Nos. 8 and 9) consequently had residual pelvic intravenous tumors. Except for 1 patient’s tumor (No. 11), none of the tumors adhered to the RA and IVC, and all could be pulled out through the incision.

For these 13 patients, the mean operation time was 370.8 ± 111.0 min, the mean intraoperative blood loss was 992.3 ± 994.5 mL, and the mean total hospital stay was 23.5 ± 7.7 days. As shown in Table 3, the total operation time (286.5 ± 71.9 min vs. 443.1 ± 84.4 min; *P* = 0.004) and intraoperative blood loss (483.3 ± 213.7 mL vs. 1429.2 ± 1208.0 mL; *P* = 0.020) in the Type A group were significantly less than those in the Type B group. The total hospital stay of the two groups was similar.

**TABLE 3:** Comparison of operation data between Type A and Type B groups.

	Type A surgery procedure (*N* = 6)	Type B surgery procedure (*N* = 7)	*P*-value
Operative time (min)	286.5 ± 71.9	443.1 ± 84.4	0.004
Operative blood loss (ml)	483.3 ± 213.7	1429.2 ± 1208.0	0.020
Hospital stay (day)	23.5 ± 6.4	23.4 ± 9.1	0.980

### Pathology results

In the postoperative pathological examination, most tumors in the IVC and ovarian veins were gray and white, firm, and tough rope-like substances.

HE staining showed that the lesion consisted of relatively consistent spindle cells; the clustered small vessels in the tumor were similar to granulation tissue, and some were accompanied by hemorrhage and small focal necrosis.

Immunohistochemical staining indicated that all tumors were positive for SMA, Desmin, and Bcl-2, negative for CyclinD1 and CD10, and diffusely positive for PR and ER. The Ki-67-positive index was 0.5%. 3∼2.2%; specifically, 12 tumors had indices less than 1%, and only 1 tumor had an index > 2%.

### Follow-up results

All patients were followed up for an average of 26.3 ± 18.8 months. One patient had local recurrence, and 1 patient died of pancreatic cancer 12 months after the operation. Eleven patients (Nos. 1, 2, 3, 4, 5, 6, 8, 9, 10, 12, and 13) were in stable condition after the operation. Two patients (Nos. 7 and 11) experienced postoperative complications. Patient No. 7 experienced pelvic floor muscle relaxation 6 months postoperatively and was later diagnosed with vesicovaginal fistula. In patient No. 11, incomplete intestinal obstruction occurred 1 month after the operation, and the symptoms were relieved 3 months later. Patient No. 3 was diagnosed with acute pancreatitis 3 months after the operation and was later diagnosed with pancreatic cancer, dying of pancreatic cancer at 12 months of follow-up.

Three months after the operation, CTV confirmed that the IVC was unobstructed and that none of the patients had filling defects. The tumor recurred in patient No. 1 at 60 months after the operation. Patient No. 1 showed symptoms of lower abdominal pain at the 60-month follow-up, and a pelvic mass was found by CTV.

## Discussion

Intracardiac leiomyomatosis is a rare type of cardiac metastatic tumor. Its histological feature is that benign leiomyomas outside the heart extend through venous channels and reach the RA. The clinical manifestation of this type of tumor is not specific (10), and consensus on the optimal approach to remove this kind of tumor is lacking. This study summarized the previous surgical experience in resecting ICL completely with a single laparotomy, which achieved good surgical results. This study provides a valuable reference for the diagnosis and treatment of this type of patient.

Clear conclusions about the etiologies of ICL are lacking. The generally accepted theories are that the smooth muscle tissue originates from the venous walls and invasive uterine myoma (11, 12). Although the histological characteristics of ICL are benign, its biological behavior tends to be malignant. Delayed diagnosis and treatment may lead to sudden death caused by venous outflow tract obstruction. The ICL can extend along the internal or external iliac vein to the CIV and IVC, through the gonadal vein to the renal vein and IVC, and finally to the RA and RV (13). In this series, 10 cases originated from the right IIV, 8 from the right CIV, 5 from the right gonadal vein, 2 from the left gonadal vein, and 2 from the left IIV and CIV. Among them, the gonadal vein and iliac vein were involved in 5 cases. Eleven cases reached the distal end to the RA, 2 reached the RV, and no case reached the pulmonary artery.

Intracardiac leiomyomatosis often lacks specific symptoms before causing heart failure, and its clinical manifestations are usually related to the scope and size of the tumor (14). RV and pulmonary valve involvement may lead to outflow tract obstruction, pulmonary embolism, and even sudden death (3). Most symptomatic patients showed palpitations, dyspnea, syncope, lower limb edema, abdominal pain, and chest pain. Other manifestations, such as cough, chest tightness, headache, shock and vomiting, are occasionally reported (15–17). In our analysis, the most common symptoms were abdominal symptoms. We did not observe any symptoms caused by circulatory disturbance or rare symptoms, such as lower limb venous thrombosis and Budd-Chiari syndrome (18).

Surgical resection is adequate for removing ICL, and many surgical strategies have been used to this end. A classification system has been described to select the best surgical procedure (19). Since the successful resection of ICL was first reported in 1982, most studies have focused on case reports, in which two-stage procedure resection is considered an effective treatment, but it increases the physiological burden of patients (6, 7, 20). A one-stage operation, in which sterno-laparotomy is also widely used in the surgical treatment of this type of tumor, still faces a high risk of complications because of its severe trauma (6, 7, 9). This study reported the most extensive single-center study of IVL. One-stage resection, hysterectomy, and bilateral salpingo-oophorectomy simultaneous operations were preferred (21). Some centers have attempted to use less invasive surgery, but relevant surgical guidelines for this approach are not available, and the type of patients suitable for less invasive surgery has not yet been identified (8, 20, 22). In our opinion, the patients who only need a single laparotomy can be divided into types A and B according to the size of the tumor and the extent of involvement. If the tumor in the IVC is larger than the maximum diameter of the infrarenal IVC, it is classified as type B and requires extensive venotomy. If the tumor is smaller than the maximum diameter of the infrarenal IVC, it is classified as type A and requires only a short venotomy. According to our experience, in patients with types A and B, the maximal diameter of the intracardiac mass is usually very small, and the mass does not adhere closely to the IVC or the intima of the heart and can be removed through the distal incision; however, blindly removing the tumor carries the risk of tearing the IVL. This experience is similar to the findings of previous studies (23). If the maximum diameter of the intracardiac mass is larger than the diameter of the distal incisional vein, the mass cannot easily be removed through a short incision, and adhesions are often found in the cardiac or inferior vena cava in these patients, which require blunt dissection by enlarging the venous incision. Due to the wide incision, we believe that these patients require CPB to assist the procedure to ensure hemodynamic stability. In the past, we thought that resection was difficult in stage III and IVL patients without CPB. These patients can be refined through clinical practice and surgical experience in recent years, and a significantly larger proportion of patients can achieve complete resection of the mass through a simple abdominal incision under non-cardiopulmonary bypass (21). Bleeding can be confidently controlled by blocking bands around the venous incision. However, in type B patients, great care should be taken when extensive venotomy is performed, which causes more bleeding than in type A patients. Therefore, cell salvage devices should be routinely applied. CPB should be instituted when necessary to maintain hemodynamic stability.

Intraoperative TEE application to determine the mass movement and the residual condition after resection has become mandatory. The key to the procedure is to control the bleeding from the venous incision. Depending on the incision site, we usually use two vein block methods, the purse-string suture and a blocking band covered by a silicone tube. The key to the purse-string suture is to use a silicone tube to tighten near the suture and add a blocking band covered with a silicone tube at the proximal-distal end of the incision. The tumor usually has good elasticity, and a small incision is therefore better than a large incision to allow the entire tumor to be removed while gradually tightening the suture to reduce the bleeding at the incision. For ICL of multiple sources, a double incision is necessary. For example, if the iliac vein is adherent to the mass, bleeding can also be controlled around the iliac vein incision using purse-string sutures with silicone tubing blocking tape based on adequate exposure to the internal and external iliac veins. In addition, CPB should be a backup option in any patient with severe intraoperative adhesions prevent the removal of the tumor from the venous incision alone. In this study, Type B patients had a longer operative times and more intraoperative bleeding than Type A patients due to more extensive IVC incisions, but the total hospital stay did not significantly differ between the two groups, which shows that smaller venous incisions should be employed whenever possible.

Immunohistochemical staining of the postoperative specimen showed Desmin-, Bcl- 2-, and SMA-positive cells, indicating that the tumor originated from smooth muscle cells. In contrast, cyclin D1 and CD10 were negative, implying that the tumor did not originate from endometrial stromal cells. The theory of the leiomyoma origin of IVL was confirmed. Morphologically, we observed that although IVL showed small focal necrosis, cell-rich foci, and epithelioid structures, nuclear schwannomas were rare, while apparent cellular anisotropy and pathological nuclear schwannomas of leiomyosarcoma did not occur.

In a review report including 192 ICL patients, follow-up data were obtained from 111 patients, including 75 patients without recurrence in the complete tumor removal group, and the postoperative follow-up was up to 12 years. In the incomplete removal group (*n* = 36), the recurrence rate was 33.3%, and the postoperative survival rate was 11.1% (24). Data from 10 years post-IVL follow-up at our center showed a recurrence rate of 31.0% among 58 patients during a median follow-up period of 11.5 months; our data also suggest that recurrence is associated with preoperative tumor extent and large vein involvement (25). A total of 13 patients were included in this study, among whom 3 patients had incomplete removal after surgery. All but one patient, who experienced recurrence of a pelvic mass 5 years after surgery, were free of recurrence. The low recurrence rate may be associated with the high rate of complete removal, and all patients underwent complete hysterectomy. Due to a lack of randomized controlled data, recommendations for postoperative antiestrogen therapy remain controversial. No recent studies have shown clear evidence for the efficacy of estrogen antagonists in the treatment or recurrence prevention of IVL (25). We do not routinely apply these agents postoperatively.

The main limitations of this study are as follows. First, ICL is a rare disease, and the sample size was small. Second, this work was a retrospective study. The purpose of this study was to further analyze patients undergoing non-open cardiac surgery and to propose a new surgical standard. Although many centers treat patients with ICL, clear surgical criteria are lacking. The data may be biased due to the small sample size, and the applicability of our proposed new criteria to other centers needs to be confirmed with more cases and longer follow-ups.

## Conclusion

One-stage surgical resection of the tumor through a single laparotomy is feasible for ICL patients, which reduces the impacts of thoracotomy and extracorporeal circulation on the patient. Different surgical resection strategies can be chosen depending on the morphology and extent of tumor involvement. Patients who undergo short venotomy can obtain shorter operative times and less blood loss than patients with extensive venotomy.

## Data availability statement

The data analyzed in this study is subject to the following licenses/restrictions: The raw data supporting the conclusions of this article will be made available by the authors, without undue reservation. Requests to access these datasets should be directed to CW.

## Ethics statement

The studies involving human participants were reviewed and approved by the Ethics Committee of Peking Union Medical College Hospital. The patients/participants provided their written informed consent to participate in this study.

## Author contributions

CW, JS, and XM were responsible for the data acquisition, analysis, and manuscript drafting and revising. YZ and GM were responsible for data collection and manuscript revision. NC, DC, ZL, XS, and KL were responsible for manuscript revision. BL was responsible for the study design, manuscript revision, and final approval of publication. All authors contributed to the article and approved the submitted version.
